# Case of Ovarian Tumor; Excision; Death

**Published:** 1857-09

**Authors:** M. Kempf

**Affiliations:** Ferdinand, Indiana


ART. IV.—*Case of Ovarian Tumor; Excision; Death.* By M. KEMPF, M.D., of Ferdinand, Indiana.

IN January last I was requested to see a lady, who had been for some time the subject of an abdominal enlargement. She was thirty-five years of age, the mother of four children; the eldest eighteen, and the youngest seven years old, and much emaciated. Her countenance was expressive of great anxiety; respiration hurried and difficult; skin blanched; pulse ninety and feeble; tongue moist and clean. She gave me to understand that about four years previous, she noticed a swelling or lump, about the size of the fist, in her abdomen, situated between the umbilicus and pubes somewhat on the right side, which gradually increased in size until it occupied the whole space between the pelvis and thorax, greatly impeding respiration, and oftentimes producing a distressing sense of suffocation. Her general health began to decline about the same time, and her catamenia, continuing for some months after, gradually disappeared entirely.

She had used from time to time hydragogue cathartics, but experiencing little or no benefit, she had latterly abandoned them.

After a careful examination, I diagnosed the case to be one of ovarian dropsy, but determined, before resorting to more violent measures, to give the class of remedies just mentioned a fair trial, and prescribed elaterium, grs. 1½; gamboge, grs. xiii; ext. jalap, ℈i; ext. hyoscyamus, grs. x; to be made into twelve pills, of which three were to be taken at bedtime, and one every hour the following morning, until large watery dejections should be produced.

No benefit resulting from this course, after some days, I persuaded the patient to allow the water to be drawn off; to which she having consented, I introduced a trocar, midway between the umbilicus and pubes, and a little to the right of the median line; but, to my astonishment, no water made its appearance. Confident that I had not made an error in diagnosis, I entered the instrument again, about an inch below the umbilicus, when a limpid stream of water flowed through the canula, much to the patient's relief.

In three weeks the water had reaccumulated, and I was again requested to draw it off, which I did, and injected tinct. iodine, ℥i; ordered opium, grs. iii, and perfect rest. No unpleasant symptoms followed the operation, but no benefit was experienced.

The patient urgently requesting me to employ any other means that might offer a reasonable hope of success, I called Dr. Huber in consultation, and after a careful consideration of the case, we concluded that extirpation was the only hope. Accordingly, on the 13th of April, the fluid having been drawn off by a trocar, the patient allowed to rally from this procedure, and chloroform administered, I made an incision along the middle line of the abdomen, from the umbilicus to the pubes. The cavity was first opened near the umbilicus, and enlarged from above downwards. About midway of the incision, the index and middle fingers of my left hand, which I used as a director, came in contact with the tumor, adherent to the anterior abdominal wall. I tried to break up these adhesions, but failing in this, I touched them gently with the edge of the knife; they readily gave way upon farther traction, when the opening was continued on to the pubes.

At this stage of the operation the patient became very much prostrated; the pulse was gone at the wrist; the extremities became cold, and the jaws rigidly closed. Indeed, there was every appearance of immediate death; but by frictions of the surface and the internal administration of brandy, under the active direction of Dr. Huber, reaction was established. I then proceded to apply a ligature to the pedicle of the tumor, and having drawn it tightly, divided the pedicle as near the tumor as possible. But in attempting now to lift the mass from the cavity, I found it adherent behind to a coil of intestine, about six or seven inches in length. Not being able readily to break up this attachment, I made a slight incision around its circumference when it yielded without any difficulty, and no hemorrhage followed.

The wound was closed with interrupted suture and adhesive strips, supported by two compresses and a bandage. The patient was then put to bed, and warmth being applied to the extremities, reaction took place in the course of ten or fifteen minutes; and an hour after she appeared cheerful, said she had felt no pain during the operation; pulse ninety; surface of the body of a natural temperature, and respiration easy. Ordered opium, grs. iii.

At 8 o'clock in the evening of the same day the pulse was eighty; skin warm, respiration natural, but little pain in the abdomen; micturated without much difficulty. She complained however of pain in her mouth and difficulty of deglutition; and upon examination I found the buccal mucous membrane somewhat swollen. Ordered the opium to be repeated during the night, in case of much abdominal suffering.

April 14th, A.M., pulse ninety-five; aphthæ visible on tongue and inside of cheeks; vomited twice this morning; urinated without difficulty. Took six grains of opium during the night. Not much pain over the abdomen. Ordered Sulph. Morph. grs. iss; acaciæ pulv. ℈i, to be made into twelve powders, one of which to be taken every three hours. Diet, toast and tea, with orange-peel water to drink.

5 P.M. same day. No vomiting since morning: general condition much the same.

15th, A.M. Patient did not rest well during the night; pulse one hundred and five; much thirst; complains of considerable pain in abdomen; has urinated several times. Ordered laudanum sixty drops, to be repeated in doses of twenty-five drops, if pain does not subside until desired effect is produced.

16th, A.M. Slept well part of the night; much thirst; pulse one hundred and five.

5 P.M. Abdomen slightly tympanitic; increased pain. Ordered morphia powders as on the 14th, with the addition of one grain of calomel to each.

17th. Complains of abdominal distension and much pain.

Removed dressings, found the wound had united throughout its upper two-thirds, but from the lower part, where the ligature was situated, a thin sanious fluid escaped in sufficient quantity to wet the compress. Ordered anodyne as yesterday; an emollient poultice to the wound; and diet of chicken broth.

18th. Rested but little during the night; and vomited several times. Pulse frequent and feeble, abdomen tympanitic; wound presents same appearance as on yesterday. Ordered castor oil ʒij.

5 P.M. Oil not having operated, gave enema, which brought away some scybalæ. Ordered tinct. opii. gtt. 60.

19th, A.M. Slept moderately last night; pulse very frequent and feeble; countenance pale, contracted, and anxious; very much troubled with aphthæ on tongue and cheeks; abdomen tympanitic; lower portion of wound unhealthy and discharging a good deal of sanies; has urinated. Ordered nourishing diet and milk punch, and Ol. Ricini ℥ss; Ol. Terebinth gtt. xv; Mucilag. Acaciæ ℥i; one-half to be taken at once, and the remaining in three hours.

5 P.M. Had one scanty dejection from bowels; complains of great pain in abdomen. Ordered opium to be repeated.

20th. Patient much as yesterday, but evidently declining. P.M. died this evening. No autopsy.

The tumor was found on examination to be nearly solid, of a light straw color, lobate, and of an elastic fibrous structure. Imbedded in its substance were a number of bodies of a light brown and others of a darker color, varying in size from that of a bean to an acorn, ovoidal in shape, and of a caseous consistence. The weight of the mass was three pounds.

I need hardly remark that as the tumor proved to be solid, the water which I drew off necessarily came from the peritoneal cavity, and the injection of iodine entered the same. Had I not employed the latter, an error consequent upon a mistaken diagnosis, which I conceive that the most experienced might make under similar circumstances, it seems very likely that the result of the excision would have been successful.

